# Metabolic engineering of *Rhizopus oryzae* for the production of platform chemicals

**DOI:** 10.1007/s00253-012-4033-0

**Published:** 2012-04-13

**Authors:** Bas J. Meussen, Leo H. de Graaff, Johan P. M. Sanders, Ruud A. Weusthuis

**Affiliations:** 1 Fungal Systems Biology, Laboratory of Systems and Synthetic Biology, Wageningen University, Dreijenplein 10, 6703 HB Wageningen, The Netherlands; 2 Biobased Commodity Chemicals, Wageningen University, Bornsesteeg 59, Wageningen, The Netherlands

**Keywords:** *Rhizopus oryzae*, Metabolic engineering, Metabolic pathways, Transformation, Heterologous gene expression

## Abstract

*Rhizopus oryzae* is a filamentous fungus belonging to the *Zygomycetes*. It is among others known for its ability to produce the sustainable platform chemicals l-(+)-lactic acid, fumaric acid, and ethanol. During glycolysis, all fermentable carbon sources are metabolized to pyruvate and subsequently distributed over the pathways leading to the formation of these products. These platform chemicals are produced in high yields on a wide range of carbon sources. The yields are in excess of 85 % of the theoretical yield for l-(+)-lactic acid and ethanol and over 65 % for fumaric acid. The study and optimization of the metabolic pathways involved in the production of these compounds requires well-developed metabolic engineering tools and knowledge of the genetic makeup of this organism. This review focuses on the current metabolic engineering techniques available for *R. oryzae* and their application on the metabolic pathways of the main fermentation products.

## Introduction

Fossil resources such as coal, oil, and gas are estimated to become exhausted or unattractive to mine for bulk use within the coming decades. Added to this foreseen unavailability are the current ever-increasing costs, geopolitical instability of oil-producing regions, and sustainability issues. This formed a strong incentive for the development of alternative sources of feedstock to replace fossil resources. For energy generation, alternatives are already available in the form of geothermal, water, wind, solar, and nuclear energy. For the generation of transportation fuels and platform chemicals, the only feasible alternatives are biomass-derived products (van Haveren et al. 2008). Biomass can be converted into fuels and platform chemicals using fermentation processes with the aid of microorganisms. To be able to compete with processes based on a petrochemical feedstock, these microorganisms should exhibit high product yields (gram per gram), productivities (gram per liter per hour), and product titers (gram per liter). Other prerequisites are the ability to use many carbon sources, resistance against fermentation inhibitors that are released during biomass pretreatment, the ability to grow in absence of complex growth factors, etc.

*Rhizopus oryzae* is a fungus able to produce ethanol, l-(+)-lactic acid, and fumaric acid in high quantities using sugars derived from biomass (Abedinifar et al. 2009; Guo et al. 2010; Bulut et al. 2009; Vially et al. 2010). The market for these fermentation products is large, indicating the potential of this microorganism for the production of platform chemicals. In 2000, the global production of ethanol was 17.25 billion liters (Balat 2007) and increased to a volume over 74 billion liters in 2009 (Weusthuis et al. 2011). This market is expected to increase and exceed 125 billion liters by 2020 (Demirbas 2007). The global market for l-(+)-lactic acid in 2000 exceeded 100,000 tons (Hester 2000) and is expected to increase to 259,000 tons in 2012 (Global Industry Analysts 2008). l-(+)-lactic acid is currently primarily used as food or feed acidulant, but it can also be used in the fast expanding market of renewable plastics, solvents, or oxygenated chemicals (Goldberg et al. 2006; Datta and Henry 2006) and animal feed. The market for fumaric acid is smaller but still considerable with an annual estimated volume of 90,000 tons in 2007 (Anonymous 2007). This market is also expected to increase in the coming years. Fumaric acid is currently used in the food industry directly as a pH adjuster, preservative, or flavor enhancer. Due to its structure, it can be used for the production of polyester and alkyd resins (Goldberg et al. 1991). In addition to these platform chemicals, *R. oryzae* is also used for the production of a wide range of commercially relevant enzymes. The application of *R. oryzae* in biotechnological processes has recently been reviewed by Ghosh and Ray (2011).

In order to optimize product formation in *R. oryzae*, metabolic engineering techniques should be available, enabling the production of chemicals by the introduction of heterologous genes, overexpression of homologous genes, or knocking out existing competing pathways. This review describes the current state-of-the-art molecular techniques available for pathway engineering in *R. oryzae* and how they have been employed to study and enhance the production of its main fermentation products.

## The organism *R. oryzae*

*R. oryzae* is a filamentous fungus that is classified in the order of *Mucorales* in the phylum *Zygomycota*. The genus *Rhizopus* was first established in 1820 by the description of *Rhizopus nigricans* (Ehrenberg 1820) and is known for the formation of fermentation products like ethanol, l-(+)-lactic acid, fumaric, and to a lesser extent l-(+)-malic acid. The ability to produce fumaric acid is what sets this genus apart from *Aspergillus*, *Fusarium*, and *Penicillium* (Prescott and Dunn 1959). *R. oryzae* strains are often used in Asia for food fermentation to manufacture alcoholic beverages, ragi, or tempeh, and the strains are generally regarded as safe. Nevertheless, *R. oryzae* is also known as an opportunistic human pathogen and has a high prevalence under mucormycosis infections (Roden et al. 2005). Most mucormycosis cases have an underlying illness such as an elevated serum iron level, trauma, or a weakened immune system (Ibrahim 2011).

*R. oryzae* is ubiquitous in nature and found on decaying organic material. It is able to grow on a wide range of carbon sources, e.g., glycerol, ethanol, lactic acid, glucose, mannose, fructose, sucrose, xylose, cellobiose, fatty acids, and oils (Ban et al. 2001; Maas et al. 2006; Park et al. 2004; Skory 2000; Yin et al. 1997). All mentioned sugars have been shown to be a substrate for l-(+)-lactic or fumaric acid production. Moreover, *R. oryzae* has amylolytic (Amadioha 1998), xylanolytic (Bakir et al. 2001), pectinolytic (Saito et al. 2003), and cellulolytic (Amedioha 1993; Murashima et al. 2002; Karmakar and Ray 2010) capabilities, enabling the conversion of polymeric agricultural residues. It is able to grow well at a wide temperature range (up to 40 °C) and pH range (from 4 to 9), indicating a robust behavior and widely applicable potential.

## Production of chemicals by *R. oryzae*

The first references on the ability of *Rhizopus* species to produce organic acids appeared in 1911: Saito (1911) described lactic acid production by *Rhizopus chinensis*, and Ehlich (1911) reported the production of primarily fumaric acid, together with lactic acid, succinic acid, and malic acid, by *R. nigricans* species. Studies by Takahashi and coworkers (Takahashi and Sakaguchi 1925; Takahashi et al. 1926) indicated that also other products were formed, including ethanol. Ward et al. (1936) described the production of l-(+)-lactic acid from glucose by *R. oryzae* strains, culminating to a yield of 0.62 g/g. Since then, *R. oryzae* has been extensively studied for the production of organic acids, ethanol, enzymes, and other commercially interesting products. These studies have been reviewed recently by Ghosh and Ray (2011).

As mentioned before, *R. oryzae* can efficiently produce the fermentation end products. The maximal theoretical yield for l-(+)-lactic acid production by aerobic respiration is 2 mol of l-(+)-lactic acid per mole of d-glucose; this equals 1.0 g l-(+)-lactic acid per gram d-glucose. For fumaric acid, the maximal theoretical yield is 2 mol fumaric acid per mole d-glucose consumed, which correlates to 1.3 g fumaric acid per gram d-glucose. The highest reported yield for l-(+)-lactic acid was 0.88 g/g, and 0.86 g/g for fumaric acid with ethanol as the main byproduct (Cao et al. 1996; Zhou et al. 1999; Table 1). These results also demonstrate the ability of *R. oryzae* to withstand high product and d-glucose concentrations in excess of 100 g/L. The theoretical yield is 2 mol of ethanol per mole of d-glucose (0.51 g/g d-glucose). When grown on d-glucose, the yields obtained with *R. oryzae* are close to this maximal yield. Table 1 lists some experimental data on the production of ethanol by *R. oryzae*. The current benchmark microorganism for ethanol production on d-glucose is *Saccharomyces cerevisiae*. The main disadvantage of wild type *S. cerevisiae* for ethanol production is the inability to utilize pentose sugars, which are present in hemicelluloses hydrolysates. *R. oryzae* can grow on many carbon sources including C5 sugars and has low growth requirements. Furthermore, it is able to tolerate the inhibitors present in acid hydrolysates of lignocellulosic biomass (Karimi et al. 2005; Millati et al. 2005), and is able to—although at slow rate—utilize cellulose and hemicellulose directly (Skory et al. 1997).

**Table 1:** Literature data on the main fermentation end product production by *Rhizopus oryzae* strains

Product	*R. oryzae* strain	Reactor type	Sugar consumed (g/L)	Final product titer (g/L)	Yield (g/g)	Reference
l-(+)-lactic acid	NRRL 395	ALB	120	105	0.87	Park et al. (1998)
	ATCC 52311	ALB	94	83	0.88	Zhou et al. (1999)
	GY 18	SF	160	115	0.81	Guo et al. (2010)
Fumaric acid	*Rhizopus arrhizus* 2582	STR	130	103	0.79	Rhodes et al. (1961)
	ATCC 20344	RBC	108	93	0.86	Cao et al. (1996)
	*R. arrhizus* NRRL1526	SF	120	98	0.82	Kenealy et al. (1986)
Ethanol	CCUG 28958	SF	50	21	0.42	Millati et al. (2005)
	CCUG 22420	SF	50	22	0.44	Millati et al. (2005)
	CCUG 18663	SF	50	19	0.38	Millati et al. (2005)
	NRRL1501	SF	50	25	0.50	Skory et al. (1997)
	NRRL2625	SF	50	25	0.50	Skory et al. (1997)
	NRRL395	SF	50	19	0.38	Skory et al. (1997)

The *R. arrhizus* strains are currently classified as *Rhizopus oryzae* strains

*ALB* air lift bioreactor, *SF* shake flask, *STR* stirred tank reactor, *RBC* rotary bed contactor

## Genetic diversification of *R. oryzae* strains

*R. oryzae* strains can be divided into two types based on the primary organic acid produced when grown on d-glucose (Oda et al. 2003). One group produces primarily l-(+)-lactic acid, while fumaric and l-(+)-malic acid are the main fermentation products of the other group. To obtain information on this division, an analysis of the lactate dehydrogenase (LDH)-encoding genes and proteins was performed. It was determined that *R. oryzae* NRRL 395 has two NAD-dependent isoenzymes (LDHA and LDHB) (Skory 2000). The LDHA-encoding gene was expressed during growth in the presence of fermentable carbon sources such as d-glucose, d-xylose, or trehalose. In contrast, the LDHB-encoding gene was expressed on non-fermentable carbon sources such as ethanol, glycerol, and lactate (Skory 2000). A relationship between the l-(+)-lactic acid production and the LDH-encoding genes was found by Saito et al. (2004). Strains that produced l-(+)-lactic acid contained both *ldhA* and *ldhB* and were classified as type I strains. The fumaric and l-(+)-malic acid-producing strains contain only *ldhB* and were classified as type II strains. After sequence analysis of the various genes and markers from the two different types, it was determined that they were phylogenetically distinct. On the basis of these results, it was proposed to reclassify the strains which produce predominantly fumaric and l-(+)-malic acid as *Rhizopus delemar* (Abe et al. 2007), since this was the first name given to a *Rhizopus* strain belonging to the type II strains (Hanzawa 1912). The proposed reclassification of *R. delemar* for type II strains is not widely used in literature. As a result of this, the *R. delemar* strains will still be addressed as *R. oryzae* in the review.

## Genome analysis

In 2004, the genome of *R. oryzae* strain 99-880 (a type II strain) was published. This formed a great contribution to this research field and gave new insights for molecular techniques. This strain was the first organism to be sequenced in the polyphyletic basal lineage described as the *Zygomycetes*. It has an unusual high degree of gene duplication, which was analyzed by Ma et al. (2009). The genome of *R. oryzae* 99-880 is 45.3 Mbp in size and 20 % comprises of transposable elements. In total, 13,895 protein coding genes were predicted, which did not overlap with transposable elements. After analysis of the duplicated gene pairs and their common phylogenetic order, the conclusion was drawn that an ancestral whole-genome duplication event occurred. This event—in combination with recent gene duplications—resulted in a two- to tenfold increase in gene families related to pathogen virulence, fungal-specific cell wall synthesis, and signal transduction. This whole-genome duplication allows for growth under a wide range of adverse conditions. This can include host immune defense response and can explain the high prevalence of *R. oryzae* strains in mucormycosis infections (Roden et al. 2005). As a result of the whole-genome duplication, considerable difficulties are encountered in pathway modifications by gene knockout or silencing strategies.

## Transformation of fungi belonging to the *Mucorales*

Currently, there are several transformation systems developed for organisms in the order of *Mucorales*. These include *Absidia glauca* (Wöstemeyer et al. 1987), *Mucor circinelloides* (van Heeswijck and Roncero 1984), *Mucor miehei* (Monfort et al. 2003), *Parasitella simplex* (Burmester et al. 1992), *Phycomyces blakesleeanus* (Revuelta and Jayaram 1986), *Rhizopus niveus* (Yanai et al. 1990), and *Rhizomucor pusillus* (Wada et al. 1996). In general, the main bottleneck in heterologous gene expression for *Mucorales* is formed by the recombination and replication mechanisms affecting the introduced DNA. The DNA introduced by transformation will remain extra chromosomal and replicate autonomously since it does not require a defined origin of replication (Revuelta and Jayaram 1986; van Heeswijck 1986). As a result, transformants usually display a mitotically unstable phenotype. In addition to autonomous replication, these plasmids will form high molecular weight concatenated structures. These structures will co-migrate with genomic DNA, resulting in incorrect conclusions regarding integration (Gonzalez-Hernandez et al. 1997; Skory 2002). To increase the likelihood of integration in *R. oryzae*, a double-strand break (DSB) was introduced in the homologous region of the plasmid used for the transformation. This increased occurrence of integration to 20 % with the remainder of the transformants still containing concatenated plasmids (Skory 2002). It was hypothesized that the concatenated plasmids were a result of the re-ligation prior to integration into the genome. This re-ligation can be the result of repair mechanisms known as non-homologous end joining (NHEJ) (Lieber 1999). For the NHEJ to occur, only a few homologous base pairs are required in the break. When the homology on both ends is larger, single-strand annealing (SSA) is the dominant method of repair. For in-depth review on DNA repair, we advise the review of Pardo et al. (2009). Selection against NHEJ has been achieved by a frame-shift mutation in the selection marker on the vector, combined with a recipient strain that already contains a mutation in the genomic selection marker (Skory 2004b). Growth can only be restored when the plasmid integrates by a single homologous crossover into the genome, which results in a functional selection marker and a copy containing both mutations. After transformation with this vector, only 8 % of the transformants tested displayed the prototrophic phenotype. The remainder of the transformants still contained concatenated plasmids (Skory 2004b). When the sequences of the genes involved were analyzed, it was determined that the non-functional copy on the vector was repaired instead of the genomic copy. It was hypothesized that this was due to non-crossover mechanisms like break-induce replication or by synthesis-dependent strand annealing. Another study with the goal to increase the likelihood of double strand break repair examined the effect of the DNA break on NHEJ (Skory 2005). In this experiment, a vector digested at a single site with various overhangs was added to cell free extract. After 30 min of incubation, dimers, trimers, and degradation products of the vector were observed. An extension of the incubation time to 1.5 h resulted in a reduction of dimers and trimers and the appearance of high molecular weight structures. Upon transformation of spores with vectors containing either a 5′ or 3′ overhang, no difference was observed in the restoration of prototrophic growth. In the same study, a vector was designed which selected for integration into the genome. This vector contained a truncated *pyrG* selection marker containing the 5′ half of the gene. The recipient strain already contained a point mutation in the 3′ half of the genomic copy of the selection marker. Viable transformants could only be obtained when integration into the genome occurred. All of the transformants generated with the truncated selection marker that were tested contained integrated vectors, and multicopy inserts were frequently found. The efficiency of transformation with the truncated vector was 20-fold lower in comparison to a non-truncated vector; this was likely caused by the selection for a single integration event (Skory 2005).

## Metabolic engineering tools for *R. oryzae*

Several methods have been applied to alter the genome of *R. oryzae* and its transcription. These methods were based on the introduction of foreign DNA or random mutagenesis.

### Random mutagenesis of *R. oryzae*

Random mutagenesis is a powerful tool to disrupt gene functionality or to increase the productivity of metabolic processes. This has been accomplished in *R. oryzae* by chemical mutagenesis, for example with *N*-methyl-*N*′-nitro-*N*-nitrosoguanidine to generate auxotrophic mutants (Skory et al. 1998) or with diethyl sulfate to increase l-(+)-lactic acid production (Bai et al. 2004). Random mutagenesis has also been performed by radiation with UV light, gamma radiation with ^60^Co (Bai et al. 2004), or by low energy ion implantation (Ge et al. 2004). The downside of this technique is formed by the risk of generating multiple mutations. Also, considerable amount of time is required for screening and subsequent selection rounds. To screen the mutants for desired traits, efficient screening methods are required; an example of such a method is the screening method developed by Huang et al. (2010) to screen for mutants with a higher acid production.

In this study, *R. oryzae* spores were mutagenized with UV radiation, and an increase in the acidification was screened on agar plates by the color change of a pH indicator.

### Transformation with heterologous DNA

Next to the previously described random mutagenesis, another method to alter the genome is formed by the introduction of heterologous DNA. In recent years, the knowledge regarding (heterologous) gene expression in *R. oryzae* increased tremendously, and three transformation systems were described. One of the systems was based on DNA transfer by the microorganism *Agrobacterium tumefaciens* (Michielse et al. 2004). *A. tumefaciens* has the ability to transfer a part of its DNA called transfer DNA (T-DNA) to a broad range of hosts. For heterologous expression, this T-DNA is altered to contain the gene of interest. Upon transformation of biomass, this T-DNA integrates into the chromosome. In a proof of principle study for genetic modification by Michielse et al. (2004), an auxotrophic strain of *R. oryzae* (COM1291) was used as target organism. The *R. oryzae* strain contained a mutation in orotidine-5′-monophosphate decarboxylase (*pyrG*) gene, which was complemented by the introduction of the T-DNA. Transformants could be generated using protoplasts as starting material but not with spores or germlings. In total, eight transformants were isolated that were all mitotically stable under non-selective conditions. After further analysis, it was concluded that in two transformants, a gene conversion occurred. This conversion resulted in the restoration of the function of the genomic *pyrG* copy without the introduction of additional DNA. In the remaining six transformants, an extra *pyrG* copy integrated in the genome. Interestingly, the DNA introduced in the six transformants was integrated at the same locus indicative for a hotspot for integration. No vector DNA outside the T-DNA borders or of the second gene inside these borders was detected in the genome. With this system, it was not possible to express a gene of interest, and therefore, it is not suitable for heterologous gene expression, although integration into the genome was achieved. It was hypothesized that the integration is a result of the virulence factor proteins that coat the DNA and protect it from modification events.

In the same study describing the *Agrobacterium* system, a second method to transfer DNA was described. For this method, protoplasts were generated from mycelium and transformed with vector DNA by the CaCl_2_/PEG method (Michielse et al. 2004). The vector DNA used in this study was circular or linear in order to determine if linear material increased the likelihood of integration. In contrast to the *Agrobacterium* system, none of the generated transformants had stable phenotypes, and the vector DNA remained extra-chromosomal and replicated autonomous.

The third system to introduce DNA is formed by a particle bombardment DNA delivery system. The particle bombardment system was first developed to introduce DNA in plant cells (Klein et al. 1987). It is currently the only system successfully used for heterologous gene expression in *R. oryzae*. With this system, spores are transformed using tungsten particles coated with vector DNA. For *R. oryzae*, it was first used to determine the fate of introduced DNA in a uracil auxotrophic strain derived from *R. oryzae* NRRL 395 (Skory 2002). The uracil auxotrophy was complemented by the introduction of a vector with the functional *pyrG* copy from the same strain. Over the years, this system was further improved to express homologous genes such as *ldhA* (Skory 2004a). This was followed by the expression of the green fluorescence protein (GFP) as proof of principle for heterologous gene expression (Mertens et al. 2006). In 2007, a uracil auxotrophic strain derived from *R. oryzae* 99-880, a type II strain, was transformed with the *ldhA* gene from a type I strain (Skory and Ibrahim 2007). Functional heterologous gene expression was achieved in this strain with a cyanophycin synthetase-encoding gene (*cphA*) from a cyanobacterium with the goal to produce cyanophycin in *R. oryzae* (Meussen et al. 2012). Cyanophycin has a unique structure and can be used for the production of green chemicals. Also, a xylanase-encoding gene (*xynB*) from *Aspergillus niger* was expressed in *R. oryzae* (Meussen et al., in preparation).

The vectors developed for the GFP expression contained three different promoter elements originating from the phosphoglycerate kinase 1 (*pgk1*), pyruvate decarboxylase A (*pdcA*), or glucoamylase A (*amyA*). Of these promoter elements, the *pdcA* promoter gave the strongest signal (Mertens et al. 2006) and was selected as the promoter element for all the expression constructs described up to date.

Upon analysis of the GFP-expressing transformants, it was discovered when the fluorescent signal was present in a hyphen, it was not localized in a particular organelle or at the hyphal tip but evenly distributed. After transcript analysis in these transformants, it was concluded that there was a clear correlation between transcript level and GFP accumulation. Interestingly, the copy number of the genes did not significantly influence the accumulation of protein (Mertens et al. 2006).

### Gene knockout

Next to introducing genes for the production of new enzymes, DNA has also been introduced to create gene knockouts. With a double cross-over event, genes are knocked out and as a result, the specific loss of gene function or its effect on metabolic pathway can be studied. Currently, the only example of a successful double cross-over event described in detail for *R. oryzae* was for a high-affinity iron permease-encoding gene (*ftr1*). This gene is strongly expressed in *R. oryzae* during a host infection, suggesting a role of FTR1 in the pathogenicity of *R. oryzae*. To investigate the role of FTR1, a double cross-over homologous recombination gene knockout was successfully generated in an auxotrophic mutant derived from *R. oryzae* 99-880 (Ibrahim et al. 2010). In the generated transformants, the FTR1-encoding gene was not detected under non-selective conditions. Yet, under selective pressure, the phenotypic effect in putative *ftr1* null mutants was lost, and the FTR1*-*encoding gene was once again detected. Under the selective condition, the polynucleated nature of *R. oryzae* overcame the *ftr1* null mutant's phenotype. After 14 consecutive sporulation events and single colony inoculations under non-selective condition, the gene was not detected by PCR analysis. Nevertheless, within 48 h of growth in selective medium, the gene was again amplified by PCR (Ibrahim et al. 2010). It was speculated that the *ftr1*-encoding gene might be essential for the organism in iron-depleted media, and therefore, it was impossible to obtain a null mutant.

In addition, in this paper, it was claimed that it was possible to obtain an imidazoleglycerol-phosphate dehydratase (*his3*) null mutant in this strain, although these data were not published.

### RNA interference

Another elegant method to downregulate the expression of genes is formed by RNA interference (RNAi). RNAi is a recently discovered mechanism where double-stranded RNA triggers the degradation of a homologous sequence of messenger RNA (mRNA). As a result, translation of the corresponding protein is diminished or abolished (Fire et al. 1998). For the RNAi machinery, several proteins are required: a dicer, the Argonaut, and RNA-dependant RNA polymerase (RdRP). The dicer cuts dsRNA to double-stranded short interference RNA (siRNA). Argonaut subsequently binds to the siRNA fragments and retains single-stranded RNA. The Argonaut complex recognizes the homologous sequence of the mRNA and cleaves the strand, thereby rendering the mRNA unfit for protein translation. The cleaved mRNA strands are recognized by the RdRP, which generates more siRNA, thereby increasing the severity response. For fungi, an efficient and stable method for RNAi is formed by hairpin RNA (hpRNA) expressing plasmids (Goldoni et al. 2004). In addition, synthetic siRNAs have been introduced which trigger the RNAi response. Based on the genome sequence of *R. oryzae* strain 99-880, it was predicted that this strain contains two Argonaut copies, one dicer, and five RdRP-encoding genes (Nakayashiki and Nguyen 2008). In the same study, which investigated the role of the high-affinity iron permease-encoding gene (*ftr1*), RNAi was used to silence the gene and study its effect. A construct was generated with a sense and antisense (both 450 bp) of the *ftr1* gene held apart by a spacer element (Ibrahim et al. 2010). After transcription of the construct, a hairpin structure was formed (hpRNA) which initiated the RNAi process. This method was successful in silencing the gene, and the iron uptake of the transformants generated with this construct was reduced by roughly 50 %. In addition, the pathogenicity towards mice was greatly reduced. The transformants were used to infect mice and re-isolated from the mice that succumbed to the infection and healthy mice. It was discovered that the transformants from mice that succumbed to the infection had lost the RNAi vector, and in the healthy population, the vector was still present.

Next to the hpRNA interference, siRNAs were successfully employed to silence the *ldhA* and *ldhB* genes (Gheinani et al. 2011). These genes were silenced in an effort to reduce the pyruvate flow towards l-(+)-lactic acid and thereby increasing the ethanol formation. To this end, synthetic siRNAs were designed for a region in the LDHA-encoding gene, which had the highest sequence similarity to *ldhB*. The siRNAs were 25 nucleotides long and used to transform protoplasts generated from *R. oryzae* CCUG 28958. In total, six knockdown transformants were isolated and grown in medium containing 30 g/L d-glucose. The average l-(+)-lactic acid yield for the knockdown transformants was 0.01 g/g d-glucose; this represented a decrease of 86 % in comparison to the parent strain which had a yield of 0.07 g/g. Ethanol production increased with an average of 15 % from 0.39 to 0.45 g/g. After the knockdown, the yield of glycerol, succinate, and pyruvate increased coupled to a decrease in biomass production. The effect of gene silencing with siRNA is transient and therefore not suitable for an industrial process. The results of both studies demonstrated that RNAi can effectively be used for downregulation of gene expression.

In conclusion, it is possible to alter the genome of *R. oryzae* strains by random mutagenesis and genetic modification. The main bottleneck for heterologous gene expression is formed by the difficulties to obtain genomic integration of the vector DNA. In addition, there is a lack of dominant selection markers and the absence of multiple auxotrophic markers. Therefore, more research is required to further exploit the potential of *R. oryzae* for the production of platform chemicals.

## Pathways of the main fermentation products

**Fig. 1:**
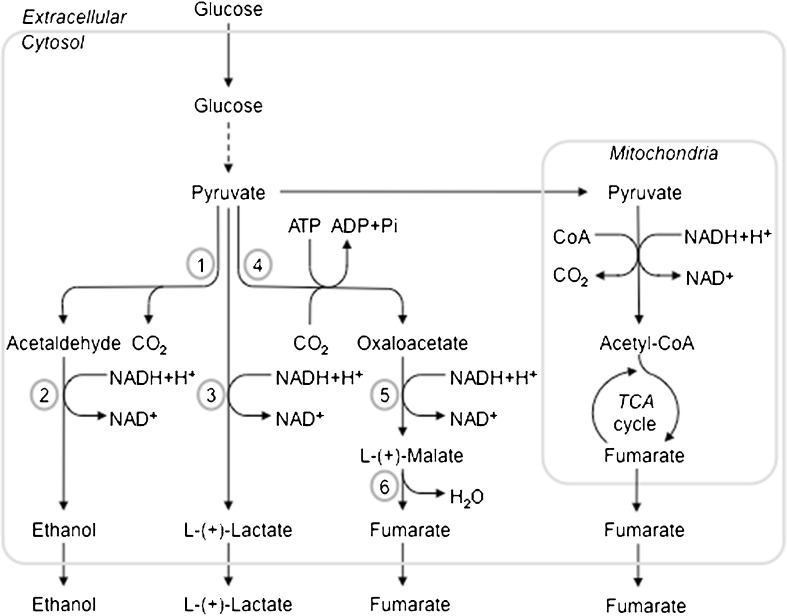
Simplified overview of the main fermentation routes with glucose as carbon source adapted from Skory and Ibrahim (2007). The *numbers* indicate key enzymes in this pathway: *1*, pyruvate decarboxylase (PDC); *2*, alcohol dehydrogenase (*ADH*); *3*, lactate dehydrogenase (*LDH*); *4*, pyruvate carboxylase (*PYC*); *5*, malate dehydrogenase (*MDH*); *6*, fumarase (*FUM*)

In *R. oryzae*, the pathways for the main fermentation products are linked to each other by the availability of pyruvate. In *R. oryzae*, all fermentable carbon sources are metabolized to pyruvate. The pyruvate is subsequently channeled to a number of pathways, including the pathways responsible for the formation of fermentation end products. This junction is named the pyruvate branch point (Fig. 1). The dissolved oxygen in the medium influences the flow of pyruvate from the branch point. Under (micro) anaerobic conditions, the carbon flow is directed towards the formation of ethanol, while under aerobic conditions, with excess of carbon substrate, the flow is directed towards organic acid production. This effect was clearly demonstrated in a study using a rotating fibrous bed reactor containing 70 g/L d-glucose (Tay and Yang 2002). The highest ethanol yield (37 % of the theoretical yield) was obtained with a dissolved oxygen (DO) of 20 %. Upon an increase to 25 % and 50 %, the ethanol yield decreased to 26 % and 15 % of the theoretical yield, respectively. These results were also demonstrated using stirred tank bioreactors containing 70 g/L d-glucose (Chotisubha-Anandha et al. 2011). The highest ethanol yield was in excess of 50 % of the theoretical yield and was obtained at a low revolution per minute. The yield decreased upon an increase in agitation or aeration rate.

The ethanol fermentative pathway is presumed to be present to allow growth for short periods of time in the absence of oxygen (Wright et al. 1996; Yoneya and Sato 1980). The production of organic acids under high sugar concentrations is presumed the result of an overflow mechanism, since cells will not encounter these high concentrations in environmental samples (Maas et al. 2008).

### Ethanol-producing pathway

The production of ethanol from pyruvate is catalyzed through the combined action of the enzymes pyruvate decarboxylase (PDC) and alcohol dehydrogenase (ADH). In *R. oryzae* NRRL 395, two PDC*-*encoding genes were detected, which were not expressed in the presence of the non-fermentable carbon source glycerol but were readily expressed after the addition of d-glucose (Skory 2003a). In addition, the absence of oxygen increased the transcript levels of these genes. When the PDC activity was compared between anaerobic and aerobic conditions, the enzyme activity was roughly three times higher under anaerobic conditions (Skory 2003a). While some knowledge is available on the PDC activity and the encoding genes, little work is performed on the characterization of ADH enzymes and encoding genes. When *R. oryzae* NRRL 395 was grown under anaerobic conditions, the ADH activity was around seven times higher in comparison to aerobic conditions (Skory et al. 1998).

In addition, it appears that that type II strains produced more ethanol in comparison to type I strains (Abe et al. 2007).This can be an effect of the absence of the l-(+)-lactic acid-producing pathway. During fermentation, the carbon sources are metabolized to pyruvate. The available pool of pyruvate is distributed over a number of pathways. When one pathway is absent, it can be assumed that more pyruvate is available for the remaining pathways, thereby increasing the amount of ethanol formed in a carbon-rich environment.

### l-(+)-lactic acid pathway

Under aerobic conditions, the main fermentation products are organic acids, presumable due to the downregulation of the PDC and ADH gene expression. l-(+)-lactic acid is produced in a single enzymatic step by a NAD^+^-dependent l-lactate dehydrogenase using pyruvate. With the aid of the particle bombardment transformation system, it was possible to gain further insight in the molecular mechanisms behind the l-(+)-lactic acid production. The LDHA*-*encoding gene from the type I strain *R. oryzae* NRRL 395 was introduced in an auxotrophic mutant derived of the type II strain *R. oryzae* 99-880 (Skory and Ibrahim 2007). The generated transformants converted more than 25 % of the starting d-glucose to l-(+)-lactic acid, whereas the recipient strain did not produce any l-(+)-lactic acid. This increase in l-(+)-lactic acid production was coupled to a reduction in the formation of biomass, fumaric acid and ethanol, thereby demonstrating a redirection of the pyruvate flow from the pyruvate branch point away from other products towards l-(+)-lactic acid. To further study the molecular mechanics of l-(+)-lactic production, the LDHB-encoding genes from the donor and recipient strains were expressed in *Escherichia coli* (Skory and Ibrahim 2007). Enzymatic analysis of both purified LDHB proteins demonstrated the ability to convert pyruvate to l-(+)-lactic acid using NADH as cofactor. Yet, the untransformed type II strain was unable to produce any l-(+)-lactic acid. It was hypothesized that the lack of l-(+)-lactic acid production was caused by tight transcriptional regulation. With Northern blot analysis, no *ldhB* transcript was detected, and a gene transcript was only detected with the more sensitive RT-PCR. The presence of very low amounts of transcript could be the cause why some type II strains are able to produce small amounts of l-(+)-lactic acid (Saito et al. 2004).

### Fumaric and l-(+)-malic acid-producing pathway

Other fermentation products originating from the pyruvate branch point are fumaric and l-(+)-malic acid. These organic acids are produced mainly via the reductive tricarboxylic acid (TCA) branch that is located in the cytoplasm. The first enzyme in this pathway is pyruvate carboxylase (PYC) located in the cytoplasm (Osmani and Scrutton 1985). In eukaryotic organisms, this enzyme is normally only present in mitochondria (Attwood 1995). PYC is a biotin-dependent enzyme and carboxylates pyruvate to oxaloacetate. In order to fully convert pyruvate to l-(+)-malic and fumaric acid, two other enzymes are required which are also located in the cytoplasm. Malate dehydrogenase (MDH) converts oxaloacetate to l-(+)-malic acid, which is hydrated (reversibly) to fumaric acid by the enzyme fumarase (FUM). The fumaric acid is subsequently transported across the cell membrane.

Of the enzymes involved in the production of fumaric acid, FUM is the best studied in *R. oryzae*. It was hypothesized by Friedberg et al. (1995) on the basis of Northern blot and primer extension analysis that *R. oryzae* has one gene (*fumR* GenBank GU013473) encoding for the enzyme FUM. However, the kinetics of the cytoplasmically and mitochondrially located enzymes is different. The different enzyme kinetics can be a result of specific conditions in the compartments or due to posttranslational modification events. It is common for eukaryotic organisms, such as rat, that both the cytosolic and mitochondrial enzymes originate from the same gene (Suzuki et al. 1992). However, Goldberg et al. (2006) suggested that there are two genes coding for the FUM enzymes present in the cytoplasm and the mitochondria. In order to determine which of the hypothesis is correct, a fumarase (*fumR* GenBank GU013473) was cloned from genomic DNA of *R. oryzae* NRRL 1526 which produced a single transcript (Friedberg et al. 1995). Antiserum produced against the *S. cerevisiae* FUM partially neutralized the enzyme activity in *R. oryzae* cell free extracts (unpublished data from Battat, Pines, and Goldberg, cited by Goldberg et al. (2006)). In order to determine whether different fumarases were present at various growth stages, the enzyme activity was determined in cell free extract from mycelium in a growth stage and in the acid-producing stage. The *K*_m_ of the FUM from the growth stage mycelium was 0.78 mmol L^−1^ for fumarate and 2.9 mmol L^−1^ for l-(+)-malic acid. The fumarase activity was not inhibited by the presence of fumaric acid. The FUM activity from the acid-producing mycelium however was completely blocked by the presence of 2 mmol L^−1^ fumaric acid. According to the authors, it was possible that a unique FUM was induced under acid-producing conditions. This enzyme would produce fumaric acid, and the reverse reaction was completely blocked by increased amounts of fumaric acid. Attempts were made by the authors to obtain this unique FUM, which had a half-life of 2 h. The gene cloned by Friedberg et al. (1995) was not further characterized. When fresh medium is inoculated with pre-grown biomass, a delay in acid fumaric acid production is observed, suggesting that two different genes are present (Roa Engel et al. 2010; Zhou et al. 2011). To further elucidate the hypothesis of the fumarase enzymes, Song et al. (2011) cloned a FUM-encoding gene (GenBank X78576) from *R. oryzae* ATCC 20344. This gene was expressed in *E. coli*, and the corresponding enzyme was analyzed. The conclusion was drawn that this FUM-encoding gene was responsible for the production of fumaric acid. The *K*_m_ for l-(+)-malic acid was 0.46 mM, and the reaction from fumaric acid to l-(+)-malic acid was blocked when the fumaric acid concentration exceeded 2 mM. These were the same observations as the unpublished results from Battat, Pines, and Goldberg cited by Goldberg et al. (2006). Song et al. (2011) then compared both the *fumR* sequence and determined that they were identical with the exception of a 15 amino acid sequence at the N-terminal end. The presence of this amino acid sequence may be responsible for the localization and activity of the enzyme. Nevertheless, the authors suggested that additional tests were required to determine if this hypothesis is correct.

It was suggested by Goldberg and Song (Goldberg et al. 2006; Song et al. 2011) that the exceptional fumarate production by *R. oryzae* is caused by the irreversibility of the reaction catalyzed by FUM at higher fumarate concentrations. However, the Δ*G*_0_′ of the conversion of l-(−)-malic acid to fumaric acid is 3.6 kJ/mol (Gajewski et al. 1985), indicating that at equilibrium, the l-(+)-malic acid concentration is higher than the fumaric acid concentration. This suggests that a dicarboxylic acid transporter with a high selectivity for fumaric acid also plays an important role in fumaric acid production in *R. oryzae*.

When *R. oryzae* is grown in a fermenter under low pH values (3.0), the cell-specific fumarate production rate is lower in comparison to a higher pH value (5.0). This can be explained by an increase in the energy requirement to maintain the internal pH, which results in the conversion of more glucose to CO_2_ and less to fumarate (Roa Engel et al. 2010).

### Conclusions and future prospects

With the ever-growing demand for platform chemicals, the increased costs of raw materials, and sustainability requirements, efficient biocatalysts are required. One of these biocatalysts is formed by the filamentous fungus *R. oryzae*. This organism is able to produce ethanol, l-(+)-lactic acid, and fumaric acid, and the ability to produce fumaric acid is what sets this genera apart from other fungi. These platform chemicals are produced in high yield on a wide range of carbon sources, in excess of 85 % of the theoretical yield for l-(+)-lactic acid and ethanol and 65 % for fumaric acid. In the cell, all the metabolic end products are formed from a common pyruvate pool, and the pathways resulting to the formation of these products are known. Recently, metabolic engineering tools have become available with which it is possible to further increase the yield. These tools consist of RNAi, random mutagenesis, and gene knockout strategies. Furthermore, it is currently possible to introduce heterologous genes, resulting in new product formation and eventually the introduction of entire pathways. To achieve the introduction of multiple genes, dominant and multiple auxotrophic selection markers should be developed. This should be coupled to additional research to gain further understanding of the fate of the DNA introduced in the cells as this rarely integrates into the genome.

While it is possible to alter the genome and to introduce heterologous DNA, the genome of *R. oryzae* itself also forms a source for interesting enzymes. Examples are the introduction of the FUMR-encoding gene in *S. cerevisiae* for the production of fumarate (Verwaal et al. 2011), the expression of *ldhA* in *S. cerevisiae* for lactic acid production (Skory 2003b), and the expression of a *R. oryzae* lipase in *Pichia pastoris* (Minning et al. 2001) and *S. cerevisiae* (Takahashi et al. 1998). The organism *R. oryzae* is a versatile organism that is already used for a wide range of applications. It is expected that this will increase in the future due to the aforementioned reasons.
